# Integrated Management Strategies for Hypertension and Diabetes in Cardiovascular Disease Prevention: A Systematic Review

**DOI:** 10.7759/cureus.111603

**Published:** 2026-06-27

**Authors:** Naresh Sen, Santosh Mohanlal Modani, Ruchu Kuthiala, Aayushi Sandipkumar Patel, Hairya Ajaykumar Lakhani, Pranjal Upadhayay

**Affiliations:** 1 Department of Cardiology, Rama Medical College and Hospital, Kanpur, IND; 2 Department of Cardiology, Dr. Santosh Cardiac Care Centre, Warangal, IND; 3 Nutrition and Psychology, School of Science, PCET’s Pimpri Chinchwad University, Pune, IND; 4 Department of Psychiatry, Dr. N. D. Desai Faculty of Medical Science and Research, Dharmsinh Desai University, Nadiad, IND; 5 Department of Internal Medicine, Smt. B. K. Shah Medical Institute and Research Centre, Vadodara, IND; 6 Department of Public Health and Medical Education, National Medical College and Teaching Hospital, Birgunj, NPL

**Keywords:** cardiovascular disease, diabetes mellitus, hypertension, integrated care, sglt2 inhibitors

## Abstract

Type 2 diabetes and hypertension frequently coexist and substantially elevate the risk of cardiovascular complications due to interconnected metabolic and vascular mechanisms. The combined presence of these conditions accelerates atherosclerosis, endothelial dysfunction, and inflammatory processes, leading to an increased incidence of adverse cardiovascular events. Existing literature primarily emphasizes individual therapeutic interventions, while limited evidence explores the combined impact of pharmacological treatments and system-level strategies in real-world clinical settings. This study evaluates evidence on therapeutic approaches and integrated care models in relation to cardiovascular outcomes among affected populations. A systematic review with narrative synthesis was conducted without meta-analysis, incorporating 10 studies identified through structured database searches, screened according to Preferred Reporting Items for Systematic Reviews and Meta-Analyses (PRISMA) criteria, and evaluated using the Newcastle-Ottawa Scale where appropriate. Across the included studies, evidence clustered around three main themes: pharmacological cardioprotection, cardiometabolic risk-factor stability, and integrated care delivery. The findings indicate that sodium-glucose cotransporter 2 (SGLT2) inhibitors and glucagon-like peptide 1 (GLP-1) receptor agonists were associated with more favorable cardiovascular profiles compared to conventional glucose-lowering therapies. Stable glycemic control and reduced hemoglobin A1c (HbA1c) variability were consistently associated with improved cardiovascular outcomes. Hypertension-related findings showed that SGLT2 inhibitors were associated with a lower risk of incident hypertension compared with dipeptidyl peptidase-4 (DPP-4) inhibitors, while multifactorial risk-factor control involving blood pressure, glycemia, lipid parameters, and smoking reduction was associated with fewer cardiovascular events. At the care-delivery level, integrated care frameworks, including HEARTS-aligned approaches, may support healthcare delivery through standardized protocols, multidisciplinary coordination, and continuous monitoring; however, protocol-level HEARTS evidence should be interpreted as implementation-planning evidence rather than proof of improved cardiovascular outcomes. These observations reinforce the importance of adopting a comprehensive, patient-centered approach that integrates both therapeutic advancements and structured healthcare systems for optimal disease management.

## Introduction and background

Hypertension and type 2 diabetes mellitus (T2DM) are highly prevalent chronic diseases that substantially contribute to global cardiovascular morbidity and mortality. Integrated management of these conditions has become an important public health priority because both disorders frequently coexist in primary care populations and require long-term, coordinated treatment strategies [1]. The implementation of combined diabetes and hypertension management has also been emphasized in broader cardiovascular disease prevention frameworks, particularly in settings where fragmented care limits risk-factor control [2]. Evidence from integrated chronic disease programs further supports the clinical value of coordinated service delivery for cardiometabolic conditions, although effectiveness varies across populations and healthcare systems [3]. Integrated care in patients with hypertension and diabetes may improve selected clinical indicators, supporting the rationale for examining combined management strategies in this high-risk population [4].

The clinical importance of this topic is strengthened by the close relationship between diabetes, hypertension, and cardiovascular disease. Contemporary cardiovascular prevention literature emphasizes that diabetes management should extend beyond glucose-lowering and should include comprehensive reduction of cardiovascular risk [5]. Lifestyle-related prevention strategies, including nutrition and physical activity, remain important components of risk reduction, although their effectiveness depends on sustained implementation and alignment with pharmacological therapy [6,7]. In the present review, methodological reporting and quality assessment were addressed separately using established review and protocol-reporting frameworks, including Standard Protocol Items: Recommendations for Interventional Trials (SPIRIT) 2013, Preferred Reporting Items for Systematic Reviews and Meta-Analyses (PRISMA) 2020, and the Newcastle-Ottawa Scale (NOS), where appropriate [8-10].

Current scientific statements recommend comprehensive cardiovascular risk-factor management for adults with T2DM, including coordinated control of glycemia, blood pressure, lipids, weight, smoking status, and lifestyle behaviors [11]. The coexistence of diabetes and obesity further increases cardiac risk through shared metabolic and structural pathways [12]. Exercise training has additional relevance in patients with T2DM and cardiovascular disease because it can improve functional capacity, metabolic control, and vascular health when incorporated into structured care [13]. Digital health technologies and monitoring tools may also support disease management by improving surveillance, adherence, and risk-factor tracking [14]. These priorities are especially relevant in regions with a high hypertension burden and limited healthcare resources, where scalable interventions are needed to reduce cardiovascular complications [15]. Mortality and vascular disease remain major concerns in both type 1 diabetes and T2DM, reinforcing the need for strategies that address multiple determinants of vascular injury [16].

Several included clinical studies provide direct evidence on pharmacological strategies for cardiovascular risk modification in T2DM. Adding glitazones or alpha-glucosidase inhibitors to metformin has been associated with lower cardiovascular risk in patients with T2DM [17]. Glucagon-like peptide-1 (GLP-1) receptor agonists have shown favorable cardiovascular safety compared with other glucose-lowering agents in real-world patients with T2DM [18]. The coexistence of T2DM and hypertension has been associated with a higher incidence of cardiovascular disease and stroke in real-world cohort data [19]. Comparative studies also support the clinical relevance of sodium-glucose cotransporter-2 (SGLT2) inhibitors. SGLT2 inhibitor use has been associated with a lower incidence of heart failure and myocardial infarction compared with dipeptidyl peptidase-4 (DPP-4) inhibitor use [20], while empagliflozin has been associated with a lower cardiovascular risk than DPP-4 inhibitors in adults with and without established cardiovascular disease [21].

Beyond medication class, the stability of metabolic control appears clinically important. Higher hemoglobin A1c (HbA1c) variability has been associated with increased major adverse cardiovascular outcomes in patients with T2DM and elevated baseline risk, suggesting that glycemic fluctuation may provide prognostic information beyond average glycemic control alone [22]. Multifactorial risk-factor control also remains central to prevention. Better control of glycemia, blood pressure, cholesterol, triglycerides, and smoking has been associated with fewer cardiovascular events and lower cardiovascular mortality in patients with T2DM [23]. SGLT2 inhibitor use has also been associated with lower incident hypertension compared with DPP-4 inhibitor use among patients with diabetes without baseline hypertension [24]. Comparative evaluation of hypoglycemic drug classes in patients with both T2DM and hypertension has shown differences in major adverse cardiovascular event (MACE) risk and safety outcomes across treatment classes [25].

System-level strategies are also relevant to this review, but they require careful interpretation. A pilot protocol has described the implementation of integrated hypertension and diabetes management using the World Health Organization HEARTS model in the Guatemalan primary care system [26]. Because this was a protocol study without completed clinical outcome data, it should not be interpreted as evidence of cardiovascular risk reduction. Its value lies in illustrating a planned implementation strategy for integrated cardiometabolic care rather than demonstrating clinical effectiveness.

Despite growing evidence on pharmacological therapies, glycemic stability, blood pressure control, lifestyle modification, and care-delivery models, the combined evidence base remains heterogeneous. Many studies evaluate individual drug classes or isolated risk factors, while fewer studies examine how therapeutic selection and structured care delivery interact in real-world cardiometabolic disease management. Therefore, this systematic review with narrative synthesis evaluates evidence on cardiovascular endpoints, hypertension-related outcomes, and integrated care approaches in adults with T2DM, hypertension, or both conditions.

Objective of the review

This systematic review with narrative synthesis evaluates pharmacological treatments, cardiometabolic risk-factor control, and integrated care strategies in adults with T2DM, hypertension, or both conditions. It aims to compare how these approaches relate to cardiovascular endpoints, hypertension-related outcomes, and implementation-relevant care delivery.

## Review

Methodology

Study Design

This study was conducted as a systematic review with narrative synthesis and without meta-analysis to evaluate cardiovascular risk-management strategies in adults with T2DM, hypertension, or both conditions. The population scope reflected the included evidence base: coexisting cardiometabolic disease, T2DM with cardiovascular or hypertension-related endpoints, incident hypertension studies, and integrated care implementation models. Meta-analysis was not performed because the included studies differed in population characteristics, study design, interventions, comparators, follow-up periods, and outcome definitions. Findings were synthesized narratively and presented according to clinically relevant subgroups.

Data Selection and Search Strategy

A systematic search was conducted in PubMed/MEDLINE, Scopus, Web of Science, Embase, and Google Scholar. The final search was conducted on May 30, 2026, and studies published from January 2015 to May 2026 were considered. Search concepts included “type 2 diabetes,” “hypertension,” “cardiovascular outcomes,” “cardiovascular endpoints,” “SGLT2 inhibitors,” “GLP-1 receptor agonists,” “risk-factor control,” and “integrated care.” These concepts were combined using Boolean operators, including AND and OR, with database-specific syntax adapted according to each platform’s indexing structure. The complete PubMed/MEDLINE search string has been uploaded to the “Files for Review” section. Applied filters included English-language, human studies, adult participants, peer-reviewed journal articles, and publication dates from 2015 onward. For Google Scholar, the first 200 results were screened because of relevance ranking and declining yield beyond this threshold. Reference lists of eligible studies and relevant background articles were also screened. Of the 33 references in the final reference list, 10 studies met the predefined eligibility criteria for synthesis; the remaining references supported background, reporting standards, risk-of-bias assessment, and discussion.

Eligibility Criteria

Eligible studies included adults with T2DM, hypertension, or both conditions and reported cardiovascular endpoints, hypertension-related outcomes, or implementation outcomes relevant to integrated cardiometabolic care. Studies of glucose-lowering therapies were eligible when they reported cardiovascular or hypertension-related endpoints. Studies evaluating incident hypertension were analyzed as a separate subgroup because they addressed hypertension prevention in high-risk diabetic populations. Integrated care protocols were included separately when they addressed structured hypertension-diabetes care delivery, but they were not treated as completed clinical outcome studies. Non-human studies, review articles, editorials, conference abstracts, studies without relevant cardiovascular, hypertension-related, or implementation outcomes, and non-English publications were excluded.

Study Selection Process

The study selection process followed PRISMA 2020 [9]. After duplicate removal, two reviewers independently screened titles and abstracts. Full-text assessment was then performed independently by two reviewers. Disagreements were resolved through discussion or consultation with a third reviewer. Ten studies met the eligibility criteria and were included in the final narrative synthesis.

Data Extraction

Two reviewers independently extracted data using a standardized framework. Extracted variables included author name, publication year, study design, country or setting, population characteristics, sample size, intervention or exposure, comparator where applicable, follow-up period, cardiovascular endpoints, hypertension-related outcomes, implementation outcomes, and key findings. Discrepancies were resolved by consensus.

Risk of Bias Assessment

Risk of bias was assessed using the NOS for observational and cohort studies [10]. The domains assessed were participant selection, comparability of study groups, and outcome assessment. Two reviewers independently performed the assessment, and disagreements were resolved by consensus or consultation with a third reviewer. The protocol study was not assessed using the NOS because it did not report completed clinical outcomes; it was evaluated separately using the SPIRIT 2013 checklist [8].

Protocol Registration

This systematic review was not registered in the International Prospective Register of Systematic Reviews (PROSPERO). The absence of protocol registration is acknowledged as a methodological limitation.

Data Synthesis

A narrative synthesis was performed because statistical pooling was not appropriate. No meta-analysis or meta-regression was conducted. Findings were grouped by population and intervention type: (1) adults with coexisting T2DM and hypertension, (2) adults with T2DM and cardiovascular or hypertension-related outcomes, (3) incident hypertension prevention studies, and (4) integrated care or implementation studies. Within each subgroup, the synthesis focused on direction of effect, consistency of findings, cardiovascular endpoints, blood pressure-related outcomes, and relevance to integrated cardiometabolic risk management. No pooled percentage reduction estimates were calculated because the included studies differed in design, populations, comparators, endpoints, follow-up duration, and reported effect measures. Findings were therefore presented narratively with direct linkage to the corresponding studies and endpoints.

Results

Study Selection

**Figure 1:**
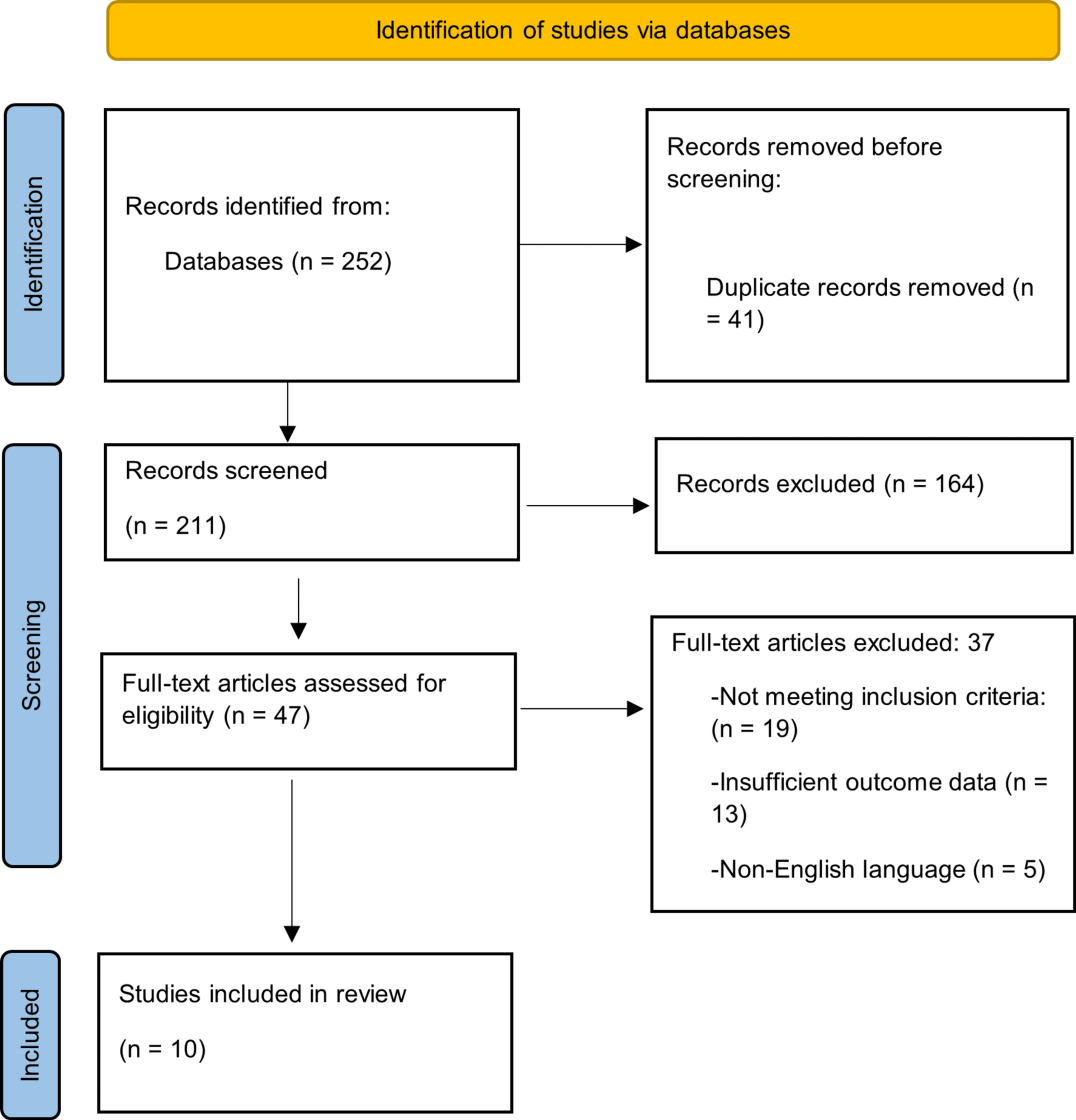
PRISMA flow diagram of the study selection process PRISMA, Preferred Reporting Items for Systematic Reviews and Meta-Analyses

A systematic database search identified 252 records related to cardiovascular outcomes, hypertension-related outcomes, and integrated cardiometabolic care in adults with T2DM, hypertension, or related cardiometabolic risk profiles. After removing 41 duplicate records, 211 records were screened by title and abstract, of which 164 were excluded. The remaining 47 full-text articles were assessed for eligibility. Of these, 37 articles were excluded because they did not meet the inclusion criteria (n=19), had insufficient outcome data (n=13), or were published in a non-English language (n=5). Finally, 10 studies met the predefined eligibility criteria and were included in the qualitative synthesis. The corrected PRISMA 2020 flow diagram in Figure 1 summarizes the identification, screening, eligibility assessment, and inclusion process.

Study Characteristics

The included evidence base consisted primarily of observational cohort and comparative effectiveness studies, along with one pilot implementation protocol. Populations varied geographically and included adults with T2DM, hypertension, or both conditions. Interventions and exposures included SGLT2 inhibitors, GLP-1 receptor agonists, DPP-4 inhibitors, other glucose-lowering drug classes, multifactorial risk-factor control, and integrated care models. Reported endpoints included cardiovascular events, heart failure, myocardial infarction, stroke, incident hypertension, cardiovascular mortality, and implementation-related outcomes. Detailed characteristics are summarized in Table 1.

**Table 1:** Key characteristics of included studies Wellmann et al. [26] was a pilot implementation protocol and did not report completed clinical outcome data. It was therefore discussed separately as an example of a planned system-level implementation strategy and was excluded from the clinical-effectiveness synthesis. T2DM, type 2 diabetes mellitus; GLP-1RA, glucagon-like peptide-1 receptor agonist; GLP-1, glucagon-like peptide-1; DPP-4, dipeptidyl peptidase-4; DPP-4i, dipeptidyl peptidase-4 inhibitor; SU, sulfonylurea; HTN, hypertension; CVD, cardiovascular disease; SGLT2, sodium-glucose cotransporter-2; HF, heart failure; EMPRISE, Empagliflozin Comparative Effectiveness and Safety; ACCORD, Action to Control Cardiovascular Risk in Diabetes; HbA1c, hemoglobin A1c; NHIS-HEALS, National Health Insurance Service-Health Screening Cohort; MACE, major adverse cardiovascular events; WHO, World Health Organization

Study	Study design	Sample size	Population/setting	Key exposure/intervention	Main cardiovascular outcome(s)	Key finding
Chan et al. [17]	Nationwide cohort observational study	26,742 patients	T2DM patients from Taiwan; mean age 56.4±11.8 years; 14,083/26,742 male patients (52.7%)	Glitazones and α-glucosidase inhibitors added to metformin	Cardiovascular risk	Addition of glitazones or α-glucosidase inhibitors to metformin reduced cardiovascular risk.
Yang et al. [18]	Nationwide population-based cohort study	3,195 GLP-1 receptor agonist stable users; matched cohorts included 1,893 GLP-1RA vs. DPP-4 inhibitor pairs, 1,829 GLP-1RA vs. sulfonylurea pairs, and 1,367 GLP-1RA vs. insulin pairs	Real-world T2DM patients from Taiwan; mean age was 49.48±11.61 years in the GLP-1RA/DPP-4i comparison, 49.34±11.64 years in the GLP-1RA/SU comparison, and 49.38±12.06 years in the GLP-1RA/insulin comparison; male proportions were 47.97%, 46.04%, and 41.70%, respectively, among GLP-1RA users in the three matched comparisons	GLP-1 receptor agonists vs. other glucose-lowering agents	Cardiovascular safety	GLP-1 receptor agonists showed favorable cardiovascular safety compared to other agents.
Liu et al. [19]	Retrospective cohort study	31,818 patients: HTN-only, n=9,653; T2DM-only, n=8,012; T2DM and HTN, n=3,592; non-T2DM/non-HTN controls, n=10,561	Chinese real-world cohort from the SuValue database; T2DM and HTN group: 1,634/3,592 male patients (45.5%) and 1,958/3,592 female patients (54.5%); age distribution in the T2DM and HTN group was 18-29 years, 31 (0.9%); 30-39 years, 139 (3.9%); 40-49 years, 553 (15.4%); 50-59 years, 927 (25.8%); 60-69 years, 1,194 (33.2%); 70-79 years, 573 (16.0%); ≥80 years, 175 (4.9%)	Presence of T2DM and hypertension	Incidence of CVD and stroke	Coexistence of diabetes and hypertension increased risk of cardiovascular diseases and stroke.
Zhou et al. [20]	Cohort study	41,994 patients	T2DM patients from Hong Kong; 58.89% male; median age 58 years (IQR: 51.2-65.3 years)	SGLT2 inhibitors vs. DPP-4 inhibitors	Heart failure and myocardial infarction	SGLT2 inhibitors were associated with a lower incidence of heart failure and myocardial infarction.
Vistisen et al. [21]	Comparative effectiveness study (EMPRISE)	85,244 empagliflozin/DPP-4 inhibitor propensity-score-matched patient pairs	Adults with T2D from Europe and Asia, with and without CVD/HF; median age ranges after matching were 61-69 years in patients with CVD, 53-64 years in patients without CVD, 62-75 years in patients with HF, and 56-65 years in patients without HF; approximately 43%-76% of empagliflozin and DPP-4 inhibitor initiators were male across countries and subgroups	Empagliflozin vs. DPP-4 inhibitors	Cardiovascular risk	Empagliflozin was associated with lower cardiovascular risk compared to DPP-4 inhibitors.
Pei et al. [22]	Secondary analysis of the ACCORD study	10,052 participants	T2DM patients with elevated cardiovascular risk from the ACCORD study; mean age 62.7±6.62 years; 6,196/10,052 male participants (61.6%)	Glycemic control and HbA1c variability	Major adverse cardiovascular outcomes	Higher HbA1c variability was associated with increased major adverse cardiovascular outcomes.
Song et al. [23]	Observational study	404,248 participants: 113,909 patients with diabetes and 290,339 subjects without diabetes	Korean NHIS-HEALS cohort; patients aged >40 years; patients with diabetes had a mean age of 63.1±9.2 years, and 53,785/113,909 were women (47.2%)	Risk factor control, including glycemia, blood pressure, cholesterol, triglycerides, and smoking	Cardiovascular events and cardiovascular mortality	Better control of cardiovascular risk factors reduced cardiovascular events, especially in patients with diabetes without cardio-renal disease.
Suzuki et al., [24]	Comparative study	18,600 individuals before matching; 5,708 matched SGLT2 inhibitor users and 5,708 matched DPP-4 inhibitor users after propensity-score matching	Japanese patients with diabetes without prior hypertension; after matching, median age was 50 years (IQR: 44-55) in SGLT2 inhibitor users and 49 years (IQR: 43-55) in DPP-4 inhibitor users; men were 4,471/5,708 (78.3%) and 4,487/5,708 (78.6%), respectively	SGLT2 inhibitors vs. DPP-4 inhibitors	Incident hypertension	SGLT2 inhibitors were associated with a lower risk of developing hypertension compared with DPP-4 inhibitors.
Wei et al., [25]	Multicenter cohort analysis	10,507 patients	T2DM patients with hypertension from two mainland Chinese electronic health record databases, Jiangsu Provincial People’s Hospital and the First Affiliated Hospital, Zhejiang University School of Medicine; age and sex distribution were not reported in the main article PDF	Different hypoglycemic drug classes added to metformin	3-point and 4-point MACE and safety outcomes	GLP-1 receptor agonists, DPP-4 inhibitors, and glinides showed lower 3-point MACE risk than insulin; DPP-4 inhibitors showed lower MACE risk than sulfonylureas in some comparisons.
Wellmann et al., [26]	Pilot study protocol	Planned sample of 100 adult patients: diabetes only, n=45; hypertension only, n=45; both diabetes and hypertension, n=10	Primary care patients in Guatemala; nonpregnant adults aged ≥18 years with T2DM, hypertension, or both; sex distribution not available because this is a protocol study	WHO HEARTS integrated hypertension and diabetes primary care model	Planned feasibility, acceptability, clinical indicator tracking, implementation outcomes, and patient-reported outcomes; no completed cardiovascular outcome data were available	The HEARTS-aligned integrated hypertension and diabetes model was included as a planned system-level implementation strategy. Because this was a protocol study, it was not included in the clinical effectiveness synthesis and was not used as evidence that cardiovascular outcomes improved or cardiovascular risk was reduced.

Risk of Bias Assessment

The methodological quality of the included observational studies was assessed using the NOS [10]. Overall, the studies showed moderate to high methodological quality, with most receiving high NOS scores across the Selection, Comparability, and Outcome domains. Selection quality was generally adequate because most studies used clearly defined populations and reliable database-based cohort identification. Comparability was the main source of variation, primarily because residual confounding may persist in observational and retrospective cohort designs despite statistical adjustment for baseline cardiovascular risk, comorbidities, and treatment-related factors. Outcome assessment was generally strong because most studies used validated clinical endpoints, registry data, or electronic health record-based outcome definitions. The study by Wellmann et al. [26] was not assessed using the NOS because it was a pilot study protocol without completed outcome data; it was assessed separately using the SPIRIT 2013 checklist [8]. Table 2 summarizes the NOS-based quality assessment of the included observational studies.

**Table 2:** Risk of bias assessment of included studies The NOS assesses nonrandomized cohort studies across three domains: selection, comparability, and outcome. The maximum score is 9 stars. Studies scoring 7-9 stars were considered high quality, 4-6 stars moderate quality, and ≤3 stars of low quality. NOS, Newcastle-Ottawa Scale; ACCORD, Action to Control Cardiovascular Risk in Diabetes

Study	Study design	Selection	Comparability	Outcome	Total NOS score	Overall quality
Chan et al. [17]	Nationwide cohort observational study	★★★☆	★★	★★★	8/9	High quality
Yang et al. [18]	Nationwide population-based cohort study	★★★☆	★★	★★★	8/9	High quality
Liu et al. [19]	Retrospective cohort study	★★★☆	★☆	★★★	7/9	Moderate quality
Zhou et al. [20]	Cohort study	★★★☆	★★	★★★	8/9	High quality
Vistisen et al. [21]	Comparative effectiveness study	★★★★	★★	★★★	9/9	High quality
Pei et al. [22]	Secondary analysis of ACCORD study data	★★★★	★★	★★★	9/9	High quality
Song et al. [23]	Observational cohort study	★★★☆	★★	★★★	8/9	High quality
Suzuki et al. [24]	Comparative cohort study	★★★☆	★★	★★★	8/9	High quality
Wei et al. [25]	Multicenter cohort analysis	★★★☆	★★	★★★	8/9	High quality

The study by Wellmann et al. [26] was not included in the NOS assessment because it was a pilot study protocol rather than a completed observational cohort study. Because protocol studies do not report completed clinical outcomes, participant follow-up results, or exposure-outcome associations, outcome-level risk of bias could not be evaluated using the NOS. Therefore, this study was assessed separately using the SPIRIT 2013 checklist, which is appropriate for evaluating the completeness and transparency of clinical trial protocol reporting. Table 3 presents the SPIRIT-based assessment of the included pilot study protocol.

**Table 3:** SPIRIT-based assessment of the included pilot study protocol SPIRIT, Standard Protocol Items: Recommendations for Interventional Trials; NOS, Newcastle-Ottawa Scale

Study	Study design	Assessment tool	Key domains assessed	Assessment summary	Overall appraisal
Wellmann et al. [26]	Pilot study protocol	SPIRIT 2013 checklist for clinical trial protocols	Administrative information, introduction, study methods, intervention description, planned outcomes, participant timeline, sample size, recruitment, data collection, monitoring, ethics, and dissemination	The protocol clearly described the study rationale, setting, eligibility criteria, intervention components, planned sample size, outcomes, implementation strategy, ethical approval, and registration. Since the study was a protocol, completed clinical outcomes and exposure-outcome associations were not available; therefore, outcome-level risk of bias could not be assessed.	Adequately reported protocol; not included in NOS assessment because no completed observational outcome data were available.

Pharmacological Interventions and Cardiovascular Outcomes

Across the included pharmacological studies, glucose-lowering therapies showed variable associations with cardiovascular endpoints in adults with T2DM, hypertension, or related cardiometabolic risk profiles. SGLT2 inhibitors and GLP-1 receptor agonists generally demonstrated more favorable cardiovascular profiles than several conventional glucose-lowering therapies, but the magnitude and direction of benefit differed across studies because of differences in populations, comparators, endpoints, follow-up duration, and reported effect measures. Zhou et al. [20] reported lower rates of heart failure and myocardial infarction among SGLT2 inhibitor users compared with DPP-4 inhibitor users, while Vistisen et al. [21] found lower cardiovascular risk with empagliflozin compared with DPP-4 inhibitors. Yang et al. [18] reported favorable cardiovascular safety for GLP-1 receptor agonists compared with other glucose-lowering agents, and Wei et al. [25] reported differences in three-point and four-point MACE risk across hypoglycemic drug classes among patients with T2DM and hypertension. Suzuki et al. [24] evaluated a hypertension-related endpoint and reported lower incident hypertension among SGLT2 inhibitor users compared with DPP-4 inhibitor users. Pei et al. [22] further showed that greater HbA1c variability was associated with increased major adverse cardiovascular outcomes, supporting the clinical relevance of stable glycemic control. Because these studies used different designs, comparators, endpoints, follow-up durations, and effect measures, their findings were not converted into pooled estimates, approximate percentage reductions, or quantitative summary figures.

Integrated Risk Management and Health System Approaches

The included studies also supported the importance of multifactorial cardiometabolic risk management. Song et al. [23] showed that better control of glycemia, blood pressure, cholesterol, triglycerides, and smoking was associated with fewer cardiovascular events and lower cardiovascular mortality, particularly among patients with diabetes without cardiorenal disease. These findings suggest that cardiovascular risk reduction in this high-risk population depends not only on glucose-lowering therapy but also on concurrent management of blood pressure, lipid parameters, smoking status, and lifestyle-related risk factors.

The HEARTS-based study by Wellmann et al. [26] was interpreted separately from the clinical effectiveness evidence because it was a pilot implementation protocol and did not report completed cardiovascular outcomes. It was therefore retained only as an example of a planned system-level strategy for integrating hypertension and diabetes care in primary care settings. The protocol described structured care components such as standardized treatment pathways, healthcare worker training, task-sharing, monitoring tools, and patient-reported outcome assessment; however, it was not used as evidence that cardiovascular outcomes improved or that cardiovascular risk was reduced. These observations support the potential relevance of system-level care models while underscoring the need for completed implementation studies with clinical outcome data.

Discussion

Cardiometabolic Risk Amplification

The current review synthesized evidence from 10 included studies to evaluate cardiovascular endpoints in adults with T2DM, hypertension, or related cardiometabolic risk profiles. Taken together, the evidence indicates that coexisting diabetes and hypertension amplify cardiovascular risk through overlapping metabolic, vascular, and inflammatory pathways. This combined disease state increases the likelihood of cardiovascular disease and stroke, emphasizing the clinical importance of early diagnosis and sustained control of cardiometabolic risk factors [19]. Rather than acting as isolated conditions, diabetes and hypertension appear to reinforce vascular injury through insulin resistance, endothelial dysfunction, vascular remodeling, and low-grade inflammation. These findings align with broader cardiovascular prevention literature identifying cardiometabolic disease as a major contributor to morbidity and mortality [11,16].

Pharmacological Cardioprotection

Against this pathophysiological background, pharmacological selection becomes a central determinant of cardiovascular risk modification. SGLT2 inhibitors and GLP-1 receptor agonists showed more favorable cardiovascular profiles than several conventional glucose-lowering therapies, including DPP-4 inhibitors and other oral agents [18,20,21,25]. These benefits were observed across clinically relevant endpoints, including heart failure, myocardial infarction, and composite cardiovascular events [20,21,25]. The cardioprotective effects of these agents are likely mediated through multiple mechanisms, including improved glycemic control, weight reduction, blood pressure lowering, natriuresis, anti-inflammatory effects, and direct vascular or myocardial actions [5,11]. These findings support treatment selection based not only on glucose-lowering efficacy but also on demonstrated cardiovascular benefit [11,17-21,25].

Glycemic Variability and Vascular Injury

The relationship between HbA1c variability and cardiovascular events provides an important mechanistic link between metabolic instability and vascular injury. Greater HbA1c variability was associated with adverse cardiovascular outcomes, even when average glycemic control appeared acceptable [22]. Fluctuating glucose exposure can activate oxidative stress pathways, impair endothelial nitric oxide bioavailability, promote endothelial dysfunction, and amplify inflammatory signaling, thereby accelerating atherogenesis and cardiovascular injury [5,11]. This mechanism strengthens the clinical argument that diabetes management should prioritize glycemic stability, not only the achievement of isolated HbA1c thresholds. Reducing glycemic excursions may therefore represent a vascular-protective strategy in high-risk cardiometabolic populations [22].

Multifactorial Risk-Factor Control

These pharmacological and metabolic findings converge with evidence supporting multifactorial risk-factor control. Simultaneous management of glycemia, blood pressure, lipid parameters, smoking, diet, and physical activity was associated with better cardiovascular outcomes than isolated risk-factor control [11,23]. This pattern reflects the interdependence of metabolic and hemodynamic injury in cardiovascular disease pathogenesis. Blood pressure control is particularly important in this context because hypertension intensifies endothelial shear stress, arterial stiffness, and target-organ damage in patients with diabetes [11,19]. Lifestyle modification further complements pharmacotherapy by improving insulin sensitivity, vascular function, weight control, and systemic inflammation [7,13]. These findings support a multidisciplinary approach that addresses multiple modifiable determinants of cardiovascular risk [11,23].

Care Delivery and Implementation Considerations

A health-system organization determines whether evidence-based strategies can be delivered consistently in routine practice. Integrated hypertension-diabetes care models emphasize standardized protocols, access to essential medicines, team-based care, monitoring systems, and structured follow-up [1,2,4]. Digital and smart health technologies may further support monitoring, adherence, and cardiovascular risk tracking when integrated into routine chronic disease care [14]. This systems perspective is particularly relevant in resource-limited settings where hypertension burden, medication access, and care-continuity barriers can undermine long-term cardiovascular prevention [15]. Together, these findings support an integrated model in which pharmacological efficacy, risk-factor stabilization, and healthcare delivery capacity operate together rather than independently. Cardioprotective therapies may have limited population-level impact if health systems cannot ensure access, monitoring, adherence, and longitudinal follow-up, while system-level interventions are most clinically meaningful when they support the delivery of evidence-based treatment [1,2,4,11].

Methodological Interpretation

The NOS assessment indicated that most included observational studies had moderate to high methodological quality, although residual bias remained possible because of non-randomized designs, confounding, and differences in population characteristics [10]. Despite these limitations, several large real-world studies supported specific elements of the synthesis: glitazones or alpha-glucosidase inhibitors added to metformin were associated with lower cardiovascular risk [17], GLP-1 receptor agonists showed favorable cardiovascular safety compared with other glucose-lowering agents [18], coexisting T2DM and hypertension was associated with increased cardiovascular disease and stroke risk [19], SGLT2 inhibitors were associated with lower heart failure, myocardial infarction, or incident hypertension risk compared with DPP-4 inhibitors [20,24], empagliflozin was associated with lower cardiovascular risk than DPP-4 inhibitors [21], HbA1c variability was associated with major adverse cardiovascular outcomes [22], multifactorial risk-factor control was associated with fewer cardiovascular events [23], and comparative hypoglycemic drug classes showed differences in MACE risk among patients with T2DM and hypertension [25].

The HEARTS-based protocol described a planned model for integrating hypertension and diabetes care through healthcare worker training, task-sharing, access to essential medications and diagnostics, standardized treatment pathways, electronic monitoring systems, and patient-reported outcome assessment [26]. Broader cardiovascular prevention literature emphasizes that risk reduction must account for prevention and treatment strategies, modifiable risk-factor management, disease-specific cardiovascular risks, vulnerable populations, psychosocial contributors, primary prevention frameworks, and integrated risk assessment approaches [27-33]. Because the HEARTS study was a pilot implementation protocol without completed clinical outcome data, it was interpreted as implementation-planning evidence rather than clinical-effectiveness evidence. Overall, the evidence supports comprehensive cardiovascular risk management in adults with T2DM, hypertension, or related cardiometabolic risk profiles, while also highlighting the need for randomized trials and completed implementation studies with standardized cardiovascular endpoints.

Limitations and Future Directions

The principal interpretive constraint of this systematic review is not the absence of evidence but the heterogeneity of the available evidence base. Most included studies were cohort, retrospective, or comparative effectiveness analyses, which provide clinically relevant real-world data but cannot establish causality with the same strength as randomized controlled trials. Observed associations may therefore reflect residual confounding, treatment-selection bias, differences in baseline cardiovascular risk, variation in medication exposure, and inconsistent adjustment for comorbidities or concurrent therapies. Differences in study design, population demographics, comparator groups, follow-up duration, and cardiovascular endpoint definitions also limited direct cross-study comparisons and precluded meta-analysis.

Although hypertension was central to the review question, the included studies primarily evaluated glucose-lowering pharmacotherapies, cardiometabolic risk-factor control, and integrated care models. Therefore, class-specific antihypertensive treatment evidence, including angiotensin-converting enzyme (ACE) inhibitors, angiotensin receptor blockers, calcium-channel blockers, beta-blockers, and diuretics, was not comprehensively synthesized. This limits the ability of the review to draw conclusions regarding preferred antihypertensive drug classes in patients with coexisting T2DM and hypertension.

This review also did not specifically synthesize emerging dual SGLT2 inhibitor/GLP-1 receptor agonist combination therapy evidence or recent cardiorenal outcome trials as separate evidence categories. As a result, conclusions regarding newer cardiometabolic treatment strategies should be interpreted in the context of the included studies and their predefined eligibility criteria. Future updates should incorporate emerging evidence on dual-class cardiometabolic pharmacotherapy, kidney-cardiovascular outcome trials, and contemporary guideline-directed approaches to combined metabolic and blood pressure management.

The inclusion of a pilot implementation protocol required separate interpretation. Because the HEARTS-aligned study did not report completed clinical outcomes, it was not used as evidence of cardiovascular risk reduction or clinical effectiveness. Its value lies in illustrating a planned health-system implementation strategy rather than demonstrating patient-level benefit. This distinction is important when interpreting the contribution of system-level care models within the broader synthesis.

Future research should move from association-based evidence toward causal and implementation-focused evaluation. Well-designed randomized controlled trials and pragmatic comparative effectiveness studies are needed to clarify the independent and combined effects of glucose-lowering therapies, antihypertensive treatment, blood pressure control, glycemic stability, and lifestyle interventions on cardiovascular endpoints. Longitudinal implementation studies are also needed to determine whether integrated care models such as HEARTS can produce sustained improvements in blood pressure control, glycemic control, medication adherence, cardiovascular events, cost-effectiveness, and patient-reported outcomes across diverse healthcare settings.

## Conclusions

This systematic review indicates a close relationship between T2DM, hypertension, and increased cardiovascular risk, supporting the need for coordinated cardiometabolic management. The included studies suggest that newer pharmacological agents, particularly SGLT2 inhibitors and GLP-1 receptor agonists, are associated with favorable cardiovascular profiles, including outcomes related to heart failure, myocardial infarction, and MACE. Stable glycemic control and multifactorial management of blood pressure, lipid parameters, smoking, and lifestyle-related risk factors also appear important for cardiovascular risk reduction. The HEARTS-aligned study should be interpreted as a planned implementation strategy rather than evidence of completed clinical benefit. Overall, cardiovascular risk management in this population requires evidence-based therapeutic selection, sustained risk-factor control, and health-system structures capable of supporting long-term monitoring, adherence, and integrated care delivery.
